# A systems biology approach toward understanding seed composition in soybean

**DOI:** 10.1186/1471-2164-16-S3-S9

**Published:** 2015-01-29

**Authors:** Ling Li, Manhoi Hur, Joon-Yong Lee, Wenxu Zhou, Zhihong Song, Nick Ransom, Cumhur Yusuf Demirkale, Dan Nettleton, Mark Westgate, Zebulun Arendsee, Vidya Iyer, Jackie Shanks, Basil Nikolau, Eve Syrkin Wurtele

**Affiliations:** 1 Department of Genetics, Development and Cell Biology, Iowa State University, Ames, Iowa 50011, USA; 2 Department of Biochemistry, Biophysics and Molecular Biology, Iowa State University, Ames, Iowa 50011, USA; 3 Department of Statistics, Iowa State University, Ames, Iowa 50011, USA; 4 Department of Agronomy, Iowa State University, Ames, Iowa 50011, USA; 5 Department of Chemical and Biological Engineering, Iowa State University, Ames, Iowa 50011, USA; 6 Center for Metabolic Biology, Iowa State University, Ames, Iowa 50011, USA; 7 Center for Biorenewable Chemicals, Iowa State University, Ames, Iowa 50011, USA

**Keywords:** *Glycine max*, Evans, seed development, seed composition, transcriptomics, metabolomics, metabolic flux, PMR, MetNet

## Abstract

**Background:**

The molecular, biochemical, and genetic mechanisms that regulate the complex metabolic network of soybean seed development determine the ultimate balance of protein, lipid, and carbohydrate stored in the mature seed. Many of the genes and metabolites that participate in seed metabolism are unknown or poorly defined; even more remains to be understood about the regulation of their metabolic networks. A global omics analysis can provide insights into the regulation of seed metabolism, even without a priori assumptions about the structure of these networks.

**Results:**

With the future goal of predictive biology in mind, we have combined metabolomics, transcriptomics, and metabolic flux technologies to reveal the global developmental and metabolic networks that determine the structure and composition of the mature soybean seed. We have coupled this global approach with interactive bioinformatics and statistical analyses to gain insights into the biochemical programs that determine soybean seed composition. For this purpose, we used **P**lant/Eukaryotic and Microbial **M**etabolomics Systems **R**esource (PMR, http://www.metnetdb.org/pmr, a platform that incorporates metabolomics data to develop hypotheses concerning the organization and regulation of metabolic networks, and MetNet systems biology tools http://www.metnetdb.org for plant omics data, a framework to enable interactive visualization of metabolic and regulatory networks.

**Conclusions:**

This combination of high-throughput experimental data and bioinformatics analyses has revealed sets of specific genes, genetic perturbations and mechanisms, and metabolic changes that are associated with the developmental variation in soybean seed composition. Researchers can explore these metabolomics and transcriptomics data interactively at PMR.

## Background

As the propagule that ensures the dissemination of plants, seeds also support human activity as one of the major products of agriculture. Both of these functions are contingent on biochemical storage reserves that are deposited within the seed during its development. Despite the vast taxonomic variation in the organ and tissue structures that are used to store these reserves, chemically they fall into three general categories: proteins, oils and carbohydrates [1]. In addition to being a major source of food, animal feed, and industrial feedstocks, these seed reserves are the primary source of carbon, nitrogen, sulfur, and energy required for seed germination and establishment of the growing seedling. During germination prior to the seedling becoming photoautotrophic, seed reserves are mobilized and their catabolism provides the emerging seedling with energy and biochemical precursors needed for growth [2,3].

There is considerable taxonomic variation in the relative ratios of seed reserves. The genetic basis for these taxonomic differences in seed composition is unclear. In each species, however, oil, carbohydrate and protein reserves are biosynthesized by the programmed expression of a metabolic network during seed development. This metabolic network converts imported photosynthetically-derived carbon (usually in the form of sucrose) and imported nitrogen (usually as amino acids) into the final proportion of protein, oil and carbohydrate [3-5].

On average, most commercial lines of soybean grown in the Midwestern states of the US are composed of 40% protein, 20% oil, 15% soluble carbohydrate, and 15% fiber [6]. Soybean seeds provide about 27% of the world's supply of oils and about 44% of the world's supply of proteins [7]. As with other plant species, soybean seeds undergo a developmentally-programmed switch in metabolism to a "seed fill" mode, such that by maturity the embryo within the seed has accumulated the three major classes of reserves. There is considerable knowledge concerning the basic biochemical processes by which imported carbon and nitrogen are converted to the final products, protein, oil and carbohydrate. A great deal remains to be learned, however, concerning the molecular, biochemical and genetic mechanisms that regulate these complex metabolic networks.

Recent developments in omics have started to provide the catalogue of genes/metabolites that are required for seed development. Analyses of mRNA abundance during seed development, maturation, and beyond are providing clues as to the global gene expression that determines final seed structure and composition in Arabidopsis [8-12], *Medicago truncatula *[13-15], *Brassica napus *[16,17], rice [18,19], barley [20], and soybean [21]. The final chemical composition of a seed is a consequence of gene expression during its development. However, this provides only a partial picture. Most genetically, developmentally, or environmentally induced alterations in biology are ultimately manifested as changes in the concentration of metabolites. Metabolomics thus can provide snapshots of the chemical composition of biological tissues over time that indicate how changes in mRNA accumulation might impact metabolism, and reveal alterations of metabolism that might result from genetic or environmental perturbations. Technological advances in metabolomics have greatly enhanced sensitivity and resolution, making it an important tool for functional genomics [22-25]. Metabolite profiling, extensively used in the design of pharmaceuticals (e.g., [26-30]), is thus providing a powerful approach to addressing plant biology [31-39]. Recently, transcriptomics and metabolomics data have been obtained to identify major developmental and metabolic transitions during soybean *var. Williams 82 *embryo development [21]. Metabolic flux mapping enables identification of the critical metabolic pathways in quasi-steady-state conditions such as seed fill [40-47], and has been applied to understand the metabolism of soybean filling [46], the effect of temperature on soybean composition [45], and the impact of light in seed fill [48].

Here, we have coupled developmental profiles of mRNA abundance with metabolomics analysis and metabolic flux within the embryo to gain insights into these integration and expression of the biochemical program that controls soybean seed development and its regulatory networks. To explore the data, and to enable others to do the same, we have deposited the transcriptomics and metabolomics data for developing soybean seeds in the public platform, PMR (**P**lant/Eukaryotic and Microbial **M**etabolomics Systems **R**esource, http://www.metnetdb.org/pmr[37]. These platforms, integrated the MetNet computational tool suite http://metnetdb.org/[38,49,50], were combined with targeted statistical analyses. The software empowers researchers to use metabolomics data and transcriptomics data of developing soybean seeds to develop hypotheses concerning the organization and regulation of metabolic networks. The combined use of these tools provides novel insights into the structure and regulation of the network that supports soybean seed development.

## Results and discussion

### Systems biology approach

Many current models of biological networks are based solely on transcript accumulation data [51], which cannot give a complete picture of cellular metabolism [52]. Thus, combined analysis of omics datasets is essential [37,53], and software is being developed to address these "big data" analyses [37]. In this study, we integrated metabolomics, transcriptomics and metabolic flux analyses to better understand the metabolic and regulatory programs that control soybean seed composition. Targeted statistical methods in R [54], combined with the MetNet systems biology platform http://www.metnetdb.org[38,49,50], were used for data analyses.

Specifically, to empower the use of soybean metabolomics and transcriptomics data in the development of hypotheses concerning the organization and regulation of metabolic networks, we modified **P**lant/Eukaryotic and Microbial **M**etabolomics Systems **R**esource (PMR: http://www.metnetdb.org/pmr[37] by incorporating soybean gene annotations from SoyBase http://www.soybase.org/[55]. Additional functional annotations of the 37,593 *Glycine max *probes were deduced via sequence homology with Arabidopsis genome annotations (TAIR10).

PMR, an interactive public database with associated analysis tools, is designed for data exploration and hypothesis formulation by researchers. In order to achieve the high power computations required for analyses of omics data, we utilize the emerging database system, NoSQL [37], which accelerates retrieval of large data from databases. Because PMR is based on a NoSQL environment [37], co-analysis of correlations with associated false discovery rate (FDR) values is quick and efficient even in big datasets such as transcriptomics data. These co-analyses can be visualized and used to postulate the genes/metabolites that may be involved in particular metabolic processes and these postulates can then be experimentally tested.

Pathways consist of a large number of entities (e.g., genes, proteins, protein complexes, enzymes, transcripts, and metabolites) and interactions among these entities. The pathways in MetNetDB are classified into different categories and visualized online http://metnetonline.org/browse_class.php. To identify over-represented pathways associated with the entities-list (e.g., differentially expressed genes in a sample), MetNet/PMR provides an "Over-representation Search" function, which ranks known metabolic pathways by the statistical significance (using Fisher's exact test) [56].

### Evans seed development

**Figure 1:**
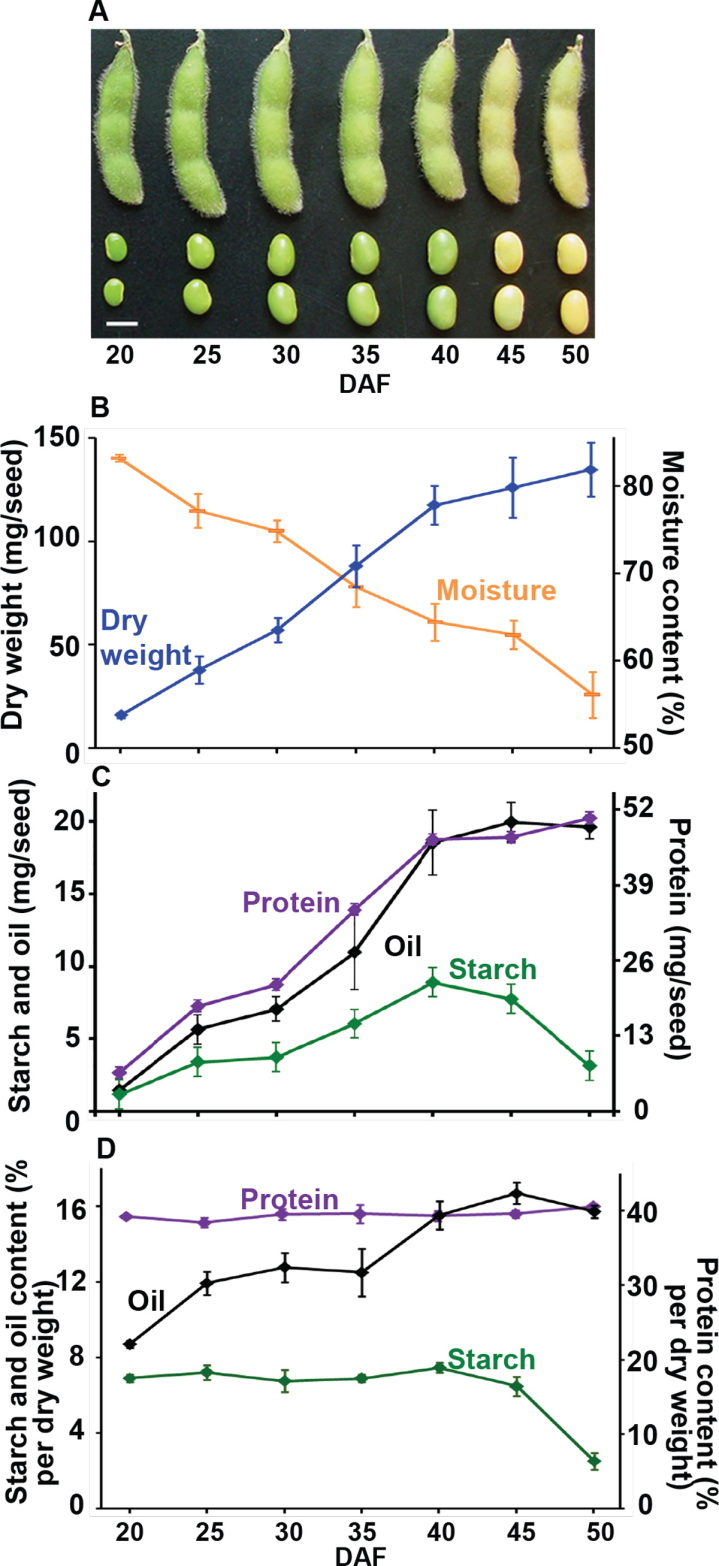
**Soybean seed development**. *Glycine max, var. Evans *plants were grown in the field and harvested at different time points from early seed fill to maturation. **A**. Pod and seed development. Bar = 1 cm. **B**. Dry weight and moisture content of seeds during development. Average ± SE, n = 3. **C**. Protein, oil and starch content (mg) per embryo during seed development. Protein, oil, and starch accumulation increases from 20 DAF and peaks at about 40 DAF. Starch content decreases rapidly after that; while protein and oil accumulation keeps stable. Average ± SE, n = 3. **D**. Protein, oil and starch content (% per dry weight) per embryo during seed development. Protein content is stable during seed development; oil accumulation increases from 20 DAF, and peaks at about 45 DAF; starch content is highest at the initial sampling times, and decreases rapidly after 45 DAF. Average ± SE, n = 3.

The development of embryos into seeds is marked by large metabolic conversions as imported photosynthate and amino acid precursors are converted to protein, oil and polysaccharide seed reserves. The timing of this developmental program is genetically predetermined and influenced substantially by environmental effects. In soybeans, this program is completed within 60 days after pollination. We used line Evans (PI 548.560), a high-yielding commercial variety previously grown in the Northern US market, to investigate the expression and accumulation profiles during this developmental timeline. Pods of a similar length containing three homogenous seeds were harvested from field-grown plants at five time points from 25 to 50 days after flowering (DAF), and seeds were placed directly into liquid N_2_. Figure 1A shows the typical morphology of the harvested pods and two of the enclosed seeds at the times of harvest. By 20 DAF, pods had reached their mature lengths and the seeds were undergoing filling (Figures 1A and 1B).

Seed filling involves increasing accumulation of protein and oil reserves, whereas starch levels decline (Figure 1C); concomitantly, these seeds gradually desiccate through this process (Figure 1B). Proportionately, oil content (% per dry weight) increases at the expense of starch content, but protein content (% per dry weight) stays relatively constant throughout this developmental program (Figure 1D). This general profile has also been reported in the oilseed Arabidopsis, which has little starch in the mature seed. However, analysis of the Arabidopsis starch-in-mature-seed *sse1 *mutant indicated that starch may be converted to oil in Arabidopsis seeds [57]. Interestingly, starch biosynthetic mutations did not increase the oil content in the *sse1 *background [58]. This suggested the decreased oil was not the result of increased carbon to starch in the *sse1 *mutant. Whether oil is generated directly from metabolism of starch in soybeans is not known, but the fact that the oil accumulation precedes the decline in starch content by >15 days (Figure 1D, 20-35 DAF) implies a more complex conversion process.

### Metabolomic determinations during seed development

To identify metabolic changes associated with seed development and storage product accumulation, metabolomic analysis was conducted on developing seeds harvested at 25, 30, 35, 45 and 50 DAF. A combination of targeted (free amino acids, esterified fatty acids, and sterols) and non-targeted profiling platforms resulted in about 400 analyte peaks, each representing one (or more) metabolic entities. Of these, 273 were above the detection limit of the analytical platforms for all replicates of at least one time point: 148 of these analytes were chemically defined (amino acids and amines (31), aromatics (7), esterified fatty acids (12), free fatty acids (12), organic acids (15), and sugars and derivatives (alcohols and acids: 41, sterols and tocopherols: 30)), and the remaining 125 are as yet unidentified.

Each metabolite's abundance data (natural log transformed and median centered) was used to identify which metabolites were differentially accumulated during seed development and q-values were calculated [59]. Interestingly, all metabolites had a q-value (FDR) < 0.17, and 173 of the metabolites had a q-value < 0.05 (Additional file 1: Table S1a). These analyses indicate that the relative abundances of the majority of the 273 metabolites varied significantly during seed development.

**Figure 2:**
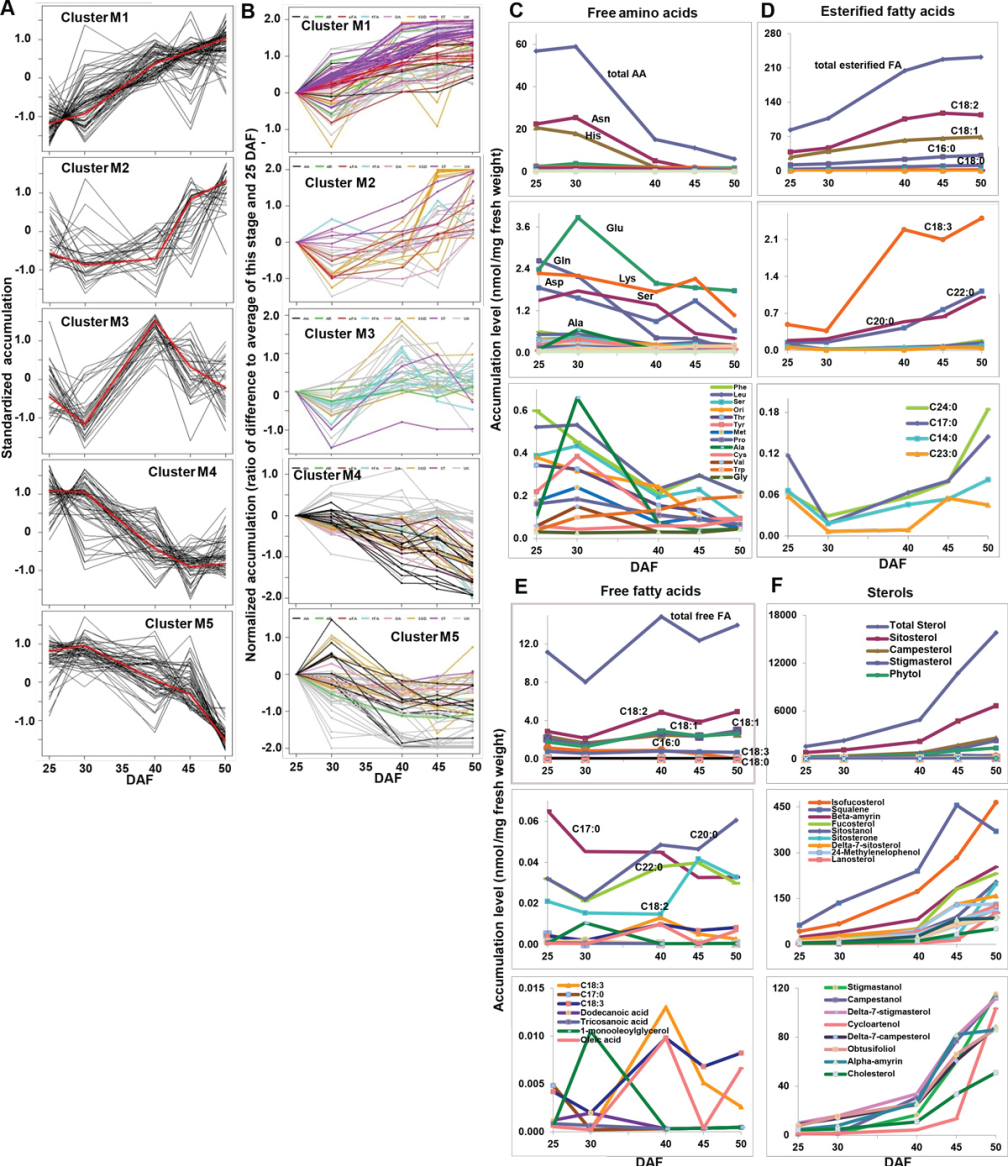
**Temporal accumulation patterns of metabolites in developing seeds**. *Glycine max, var. Evans *seeds harvested at five time points from early seed fill to maturation were analyzed by targeted and non-targeted metabolomics methods. The 273 metabolites fall into five clusters based on similar patterns of accumulation (K-medoids clustering). **A**. Standardized accumulation profiles of each cluster, with mean of 0 and standard deviation of 1; the medoid in each cluster is indicated with a red line. **B**. Clusters visualized by the ratio of difference of each stage and 25 DAF to the average of each stage and 25 DAF. Font colors-black: AA, amino acids and amines; green: AR, aromatics; red: eFA, esterified fatty acids; blue: fFA, free fatty acids; pink: OA, organic acids; orange: SGD, sugars and derivatives; purple: ST, sterols and tocopherols; grey: UK, unidentified. **C**. Temporal accumulation of free amino acids. **D**. Temporal accumulation patterns of esterified fatty acids. **E**. Temporal accumulation patterns of free fatty acids. **F**. Temporal accumulation patterns of sterols. Metabolites in **C**, **E**, and **F **were identified by targeted platforms. n = 6.

Metabolites were grouped together with similar patterns over seed development into five clusters (designated as Clusters M1-5, where M indicates it is a cluster of metabolites) (Figures 2A, B and Additional file 1: Table S1a). Each cluster showed distinguishing characteristics relative to the timing of the changes in abundance levels. In Cluster M1, metabolites steadily increased throughout seed development. Metabolites in Cluster M2 stayed at a steady basal level during early stages of seed fill, but increased around 40 to 50 DAF. Metabolite abundances in Cluster M3 peaked at 40 DAF. Metabolites in Cluster M4 reached a basal level by 45 DAF; metabolites in Cluster M5 decreased throughout seed fill.

The metabolites from the eight chemical classes of metabolites are not distributed in each cluster in proportion to their distribution in the whole group (all p-values < 0.05, Table 1 right column). Further, the metabolites in amino acids and amines, esterified fatty acids, free fatty acids, and sterols are not evenly distributed in each cluster (p-value < 0.05, Table 1 bottom row). As indicated in Figure 2B and Table 1 free fatty acids, esterified fatty acids, aromatics, and sterols are mostly grouped in Clusters M1, M2 and M3, whereas free amino acids and the organic acids are predominantly in Clusters M4 and M5. Sugars and derivatives show a mixed pattern, some increasing and other decreasing over time. We interpret these data to reflect the differential roles of different classes of metabolites during seed development, revealing the differential timing of the complex metabolic network in the seed that supports establishment of mechanisms that will enable the ensuing seedling to thrive.

**Table 1:** Metabolites were grouped into five clusters according to accumulation pattern.

	No. of Metabolites									
	**Metabolite Class^a^**									

**In Group**	**AA**	**AR**	**eFA**	**fFA**	**OA**	**SGD**	**ST**	**UK**	**Total**	**P-Value^b^**

**Cluster M1**	3	4	9	1	2	9	23	29	80	**0.00001**

**Cluster M2**	15	0	0	2	7	10	0	27	61	**0.00005**

**Cluster M3**	0	0	3	1	3	11	4	14	36	**0.03600**

**Cluster M4**	0	2	0	7	0	2	2	22	35	**0.00005**

**Cluster M5**	13	1	0	1	3	9	1	33	61	**0.00707**

**All detected**	31	7	12	12	15	41	30	125	273	

**P-Value^c^**	**7.45E-07**	0.092	**3.99E-05**	**0.023**	0.070	0.185	**1.30E-12**	0.073	**2.30E-05**	

^a^Metabolite Class: AA = Amino acids and Amines, AR = Aromatics, eFA = esterified Fatty Acid, fFA = free Fatty Acid, OA = Organic Acid, SGD = Sugars and Derivatives (alcohols and acids), ST = Sterols and Tocopherols, UK = Unclassified or Unknown.

^b^P-value, determined by Chi-square test, indicates whether the metabolites from the eight metabolite classes are distributed in each cluster in proportion to their distribution in the whole group. Significant p-values (alpha ≤ 0.05) are shown in bold.

^c^P-value, determined by Chi-square test, indicates whether the metabolites in each metabolite class are evenly distributed in each cluster. Significant p-values (alpha ≤ 0.05) are shown in bold.

Total free amino acids decreased during seed development, and their relative ratio also changed (Figure 2C and Additional file 1: Table S1b). Of the 20 free amino acids identified by the targeted platform that had a decreasing pattern during seed development (Figure 2C), at the early stages of seed fill asparagine was the most abundant, followed by histidine, glutamine, glutamate, lysine, aspartate and serine. As seeds developed, the relative proportions of asparagine, histidine, and glutamine decreased, while the relative proportions of glutamate, lysine, and aspartate increased so that by 50 DAF glutamate, lysine, and aspartate were the most abundant free amino acids. The accumulation of some amino acids (including asparagine, glutamate, serine, isoleucine, tyrosine, methionine, proline, valine, and particularly alanine) peaked at 30 DAF, while lysine, aspartate, phenylalanine, leucine, isoleucine, and methionine had a small peak at 45 DAF. Tryptophan accumulation increased throughout seed development. The concentration of free asparagine was reported to be positively correlated with protein content in maturing soybean seeds [60]; the relative high abundance of asparagine may play an important role in protein accumulation in Evans seeds.

The twelve most abundant esterified fatty acids all increased during seed development (i.e., they were in Clusters M1-3; Figure 2D and Additional file 1: Table S1c). Linoleate (18:2) was the most abundant fatty acid, followed by oleate (18:1), palmitate (16:0), stearate (18:0), and linolenate (18:3); while tricosanoic acid (23:0) was the least abundant. There was little change in the relative abundances of the individual fatty acids (Additional file 1: Table S1c).

Free fatty acid accumulation generally increased across seed development. Of the 17 free fatty acids identified by the non-targeted platform, linoleate (18:2) was the most abundant, followed by oleate (18:1), linolenic acid (18:3), and octadecanoate (22:0) (Figure 2E and Additional file 1: Table S1d). The rank of relative percent of most fatty acids compared to total fatty acids was similar; however, the rank of linolenic acid (18:3) and heptadecanoic acid (17:0) decreased when seeds were maturing.

Twenty-two of the 30 sterols, including sitosterol, campesterol, stigmasterol, phytol, and isofucosterol, increased in abundance during seed development (Figure 2F and Additional file 1: Table S1e). The relative abundance of them to total sterols was almost unchanged during seed development.

### Transcriptomic changes during seed development

In parallel to the metabolomics experiments, aliquots of the seeds were subjected to transcriptomics profiling. RNA was isolated from seeds harvested at 25, 30, 35, 45 and 50 DAF, with two biological replicates at each time point. The levels of 37,593 transcripts represented by probes on the *Glycine max *Affymetrix chip were determined. The natural logarithm of the MAS 5.0 signal was median-centered and used as the measure of expression. The analysis focused on identifying those genes that are differentially expressed during seed development. Levels of transcripts represented by 6,389 of the 37,593 probes varied among the different time points (p-value < 0.01); 2,869 of these were differentially expressed over seed development when significance was defined as q-value < 0.01.

**Figure 3:**
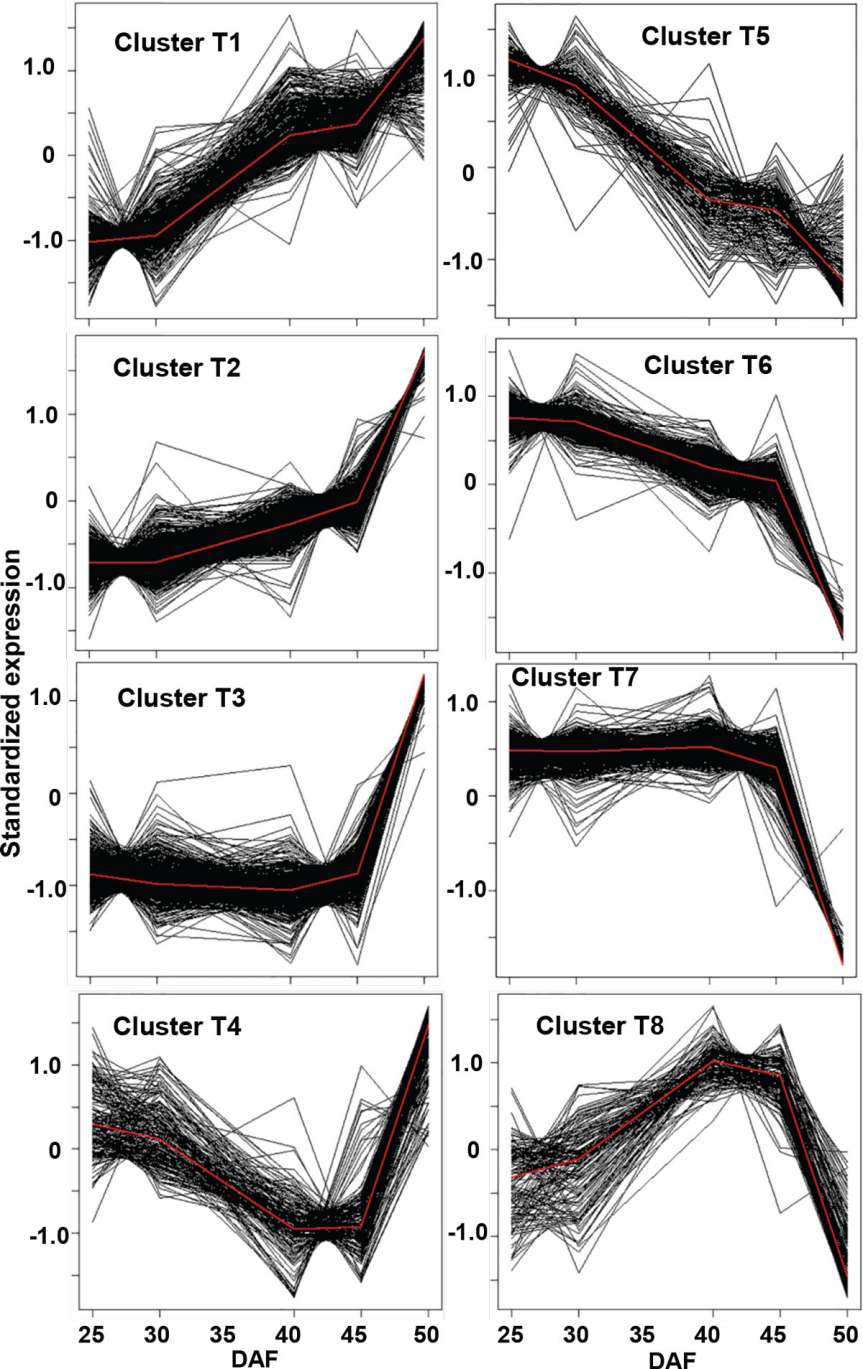
**Temporal patterns of differentially expressed genes in developing seeds**. *Glycine max, var. Evans *seeds harvested at five time points from early seed fill to maturation were analyzed by Affymetrix microarray. 2,869 of the represented genes were differentially expressed across five time points. The genes fall into 8 clusters based on similar patterns of expression (K-medoids clustering). Genes involved in starch and fatty acid synthesis are grouped in "decreasing" clusters; genes involved in starch degradation and fatty acid β-oxidation are grouped in "increasing" clusters. In addition to the genes encoding metabolic functions, clusters all contained genes of unknown function, including regulatory genes such as putative transcription factors.

These 2,869 probes that were significantly differentially expressed were grouped into eight clusters according to their expression patterns during seed development (designated as Clusters T1-8, where T indicates it is a cluster of transcripts) (Figure 3 and Additional file 2: Table S2a).

The 2,869 differentially expressed soybean transcripts were mapped to 2,451 Arabidopsis locus IDs and had 2,430 orthologous unique Arabidopsis locus IDs that provide functional annotation data (Additional file 2: Table S2a); Gene Ontology (GO) functional categorization of cellular component, biological process and molecular function http://www.arabidopsis.org/tools/bulk/go/index.jsp provides a very rough indication of the constitution of each cluster according to the GO of the matched Arabidopsis locus IDs (Additional file 2: Table S2b). Metabolic processes rank high in each cluster, followed by stress responses and developmental processes.

MetNet AtGeneSearch http://www.metnetdb.org/MetNet_atGeneSearch.htm indicated that among the 2,451 differentially expressed genes, several pathways were represented and fell into one of four categories: biosynthesis, respiration and related energetics, signaling transduction and degradation or assimilation. Particularly, represented pathways were: 1) biosynthesis of carbohydrates (sucrose and starch), lipids (fatty acid biosynthesis and elongation, linoleate and sterol synthesis), and amino acids; 2) respiration, specifically, glycolysis and the TCA cycle; 3) regulatory and signaling pathways including the AGRIS regulatory network, jasmonate (JA) biosynthesis, IAA/ethylene/gibberellin acid signaling, and regulation of gibberellin metabolism/ethylene signaling; 4) catabolism of sucrose and some amino acids (e.g., phenylalanine, glutamate, and valine) (Additional file 2: Table S2c1).

**Figure 4:**
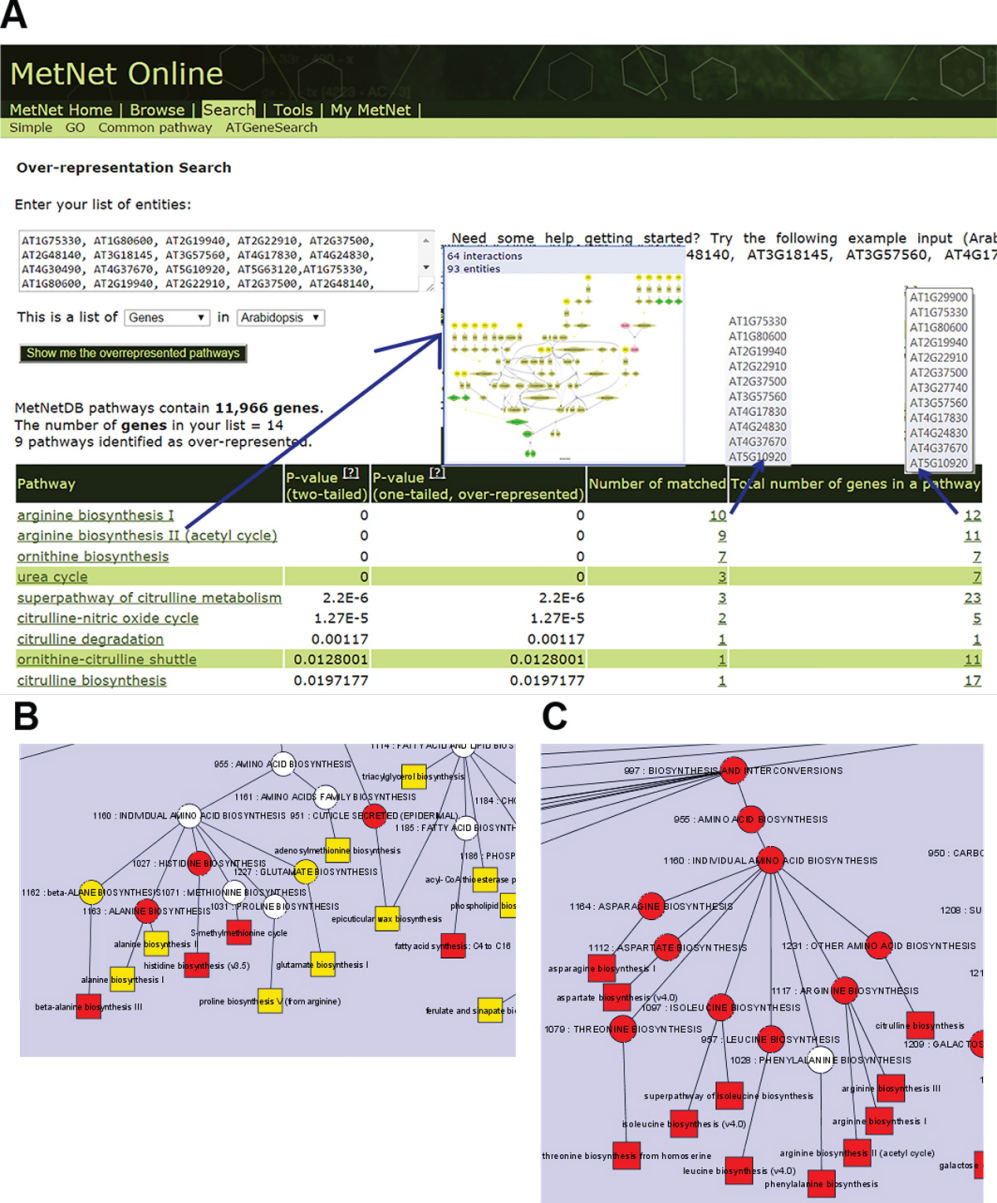
**Co-analysis of transcriptomic and metabolomic data of developing seeds**. **A**. Fisher exact test analysis of the differentially accumulated transcripts (p-value< 0.05) using MetNet showing pathways that are over-represented. Clicking on or mousing-over a pathway brings up the pathway map, and the lists of locus IDs involved in that pathway. **B**. MRPP test [61] analysis of transcriptomics data showing amino acid biosynthesis pathways that are differentially expressed across the developmental time course. **C**. MRPP test analysis of metabolomics data showing amino acid biosynthesis pathways that exhibiting significant changes in accumulation patters over the developmental time course. Oval: category of pathways; square: single pathway. Colors: q-value < 0.01, red; q-value < 0.05, orange.

MetNet's "Over-representation Search" http://metnetonline.org/searchpathway.php identifies over-represented pathways using Fisher's exact test given a user-supplied list of genes. The linoleate biosynthesis, chlorophyllide a biosynthesis, fatty acid β-oxidation, gibberellic acid biosynthesis, and 4-aminobutyrate degradation pathways are each over-represented from among all the 2,451 differentially expressed genes (Fisher's exact test p-value < 0.05) (Figure 4A and Additional file 2: Table S2c2).

Analysis of the genes from each cluster (Figure 3) shows that the pathways that most genes were involved in also belonged to one of the four groups mentioned above (Additional file 2: Table S2d); these pathways were predominantly related to the biosynthesis of carbohydrates, lipids, and amino acids. The "Over-representation Search" provided information about which pathways were over-represented in each cluster (Additional file 2: Table S2e). In particular, processes that are more enriched in particular clusters are: fatty acid beta-oxidation, jasmonic acid biosynthesis and valine and methionine degradation (Cluster T1); flavonoid synthesis, IAA signaling and lysine degradation (Cluster T2), linoleate synthesis and sorbitol degradation (Cluster T3), ricinoleate biosynthesis (Cluster T4), arginine biosynthesis, photosynthesis, glycolysis, ethylene signaling and sucrose degradation (Cluster T5), homoserine, threonine biosynthesis, glycolysis, pentose phosphate pathway, and the TCA cycle, GA and JA signaling, ammonia assimilation and glutamate degradation (Cluster T6), flavonoid biosynthesis, GA and ethylene signaling, and stress regulation (Cluster T7), and chlorophyll a biosynthesis (Cluster T8).

### Statistical analyses of the transcriptomics and metabolomics data

After an initial examination of the transcriptomics data, we focused on the 2,451 transcripts with matched Arabidopsis genes (with better annotations and pathway information than the soybean locus IDs) that showed significant changes during seed development. Using these 2,451 genes as an initial gene list, we have queried MetNetDB to identify pathways that were over-represented in the overall list, and in each gene Cluster (T1-8) as above. Here we used two statistical calculations: a) multiresponse permutation procedure (MRPP) [61]; and b) Fisher's exact test [56]. The MRPP method has the power to find pathways/categories that display significantly non-constant patterns of accumulation over time and/or categories in which the correlation structure among genes/metabolites changes over time. The MRPP method tests if a group of genes/metabolites changed expression/accumulation significantly over the seed development time series. Pathway information in MetNetDB was used to identify those over-represented pathways that contained at least two members. In contrast to the MRPP analysis [61], the Fisher's exact test is used to find groups of transcripts/metabolites over-represented within significantly changed transcripts/metabolites.

Pathways analysis of transcriptomics data by MRPP identified 273 pathways whose joint expression distribution changed significantly during development at the level of q-value < 0.05 (Additional file 3: Table S3a1). The significantly changed pathways of amino acid biosynthesis are visualized by MetNet Plug-ins for Cytoscape http://www.metnetdb.org/MetNet_fcmodeler.htm (Figure 4B). The MetNet Plug-ins for Cytoscape http://www.cytoscape.org/ are a set of Java open-source programs for visualizing and analyzing biological networks [62]. Analyses of the complex data can be linked to an interactive metabolic network display as specified by the user. This software allows for the display of a metabolic network that can be closely coupled with the other data displays and analyses; e.g., clustering, visualization, literature text processing, and data from high-throughput experiments (transcriptomics, proteomics and metabolomics) can be displayed and modeled in the context of the network graphs.

The Fisher's exact tests require at least two Affymetrix probe sets be affiliated with a pathway or category. The Fisher's exact tests identified 23 pathways/categories that are significantly over-represented among the 2,869 transcripts (Additional file 3: Table S3a2). Both MRPP tests and Fisher's exact tests identified starch and flavonoid biosynthesis (Additional file 3: Tables S3a1 and S3a2).

Gene categories defined using GO terms provided a rough idea of the results; i.e., all genes sharing any particular term were considered a category. However, not all GO terms yielded a gene category because we required that at least 2 Affymetrix probe sets be affiliated with a term before establishing a category. Both MRPP tests and Fisher's exact tests identified steroid and amino acid biosynthesis (Additional file 3: Tables S3b1-S3b6) in GO biological process.

Pathways analysis of metabolomics data by MRPP tests (Additional file 3: Table S3c1) identified 45 pathways including amino acid biosynthesis (Figure 4C), sterol and hormone synthesis. Fisher's exact tests (Additional file 3: Table S3c2) analysis identified seven pathways enriched among metabolites with differential accumulation, including arginine and sterol biosynthesis.

Pathways analysis of transcriptomics and metabolomics data by MRPP tests (Additional file 3: Table S3d) identified sterol and starch pathways as significant, as well as phospholipid, glutamate, histidine, proline biosynthesis, sucrose and starch metabolism in photosynthetic tissue, and IAA biosynthesis. These analyses indicate that genes/metabolites involved in these pathways change significantly over the time.

### Exploration of transcription factor genes that may regulate expression of the transcripts

To identify transcription factors that were candidates as regulators of expression of genes in clusters T1-8, we conducted a promoter motif analysis on the promoter regions taken as 1000 bp upstream of the "ATG" translational start-codon of each soybean transcript, using the annotation from SoyBase [55]. Within each of the promoter-regions, we identified potential motifs that regulate transcription, and compared their distribution among the promoters of the genes of each of the eight transcriptomics clusters with the promoters of all soybean genes. Fisher's exact test was used to evaluate this comparison; motifs with p-value < 0.05 are detailed in Additional file 4: Table S4. These motifs predict transcription-factor binding motifs that may control the expression of each cluster. For example, promoters from Cluster T6 genes are enriched in JA signaling and response motifs (Additional file 2: Table S2d). Particularly, 111 genes within Cluster T6 (out of 400 total), contained the promoter motif "T/GBOXATPIN2" (Additional file 4: Table S4). This "T/G-box" motif was identified in the tomato proteinase inhibitor II (pin2) and leucine aminopeptidase (LAP) gene promoters, and was thought to be involved in the JA induction of these genes; moreover, bHLH-Leu zipper transcription factors, JAMYC2 and JAMYC10 specifically recognized this motif [63]. The jasmonate biosynthesis pathway was also over-represented in this cluster. Thus, a hypothesis that we could derive from this analysis is that member(s) of the bHLH-Leu class of transcription factors in soybean, analogous to JAMYC2 and JAMYC10 in tomato and Arabidopsis, have a significant role in regulating the expression of many genes found in Cluster T6.

### Metabolic flux changes during early seed development

Carbon regulation can be evaluated by analyzing the fluxes and the flux ratios of various branch points in the reaction network [64]. Comparing flux through metabolic pathways with metabolomic and transcriptomic data can provide a novel perspective into metabolic expression resulting from genetic programing. Metabolic flux has been evaluated in soybean seeds under a number of conditions, and in several genotypes [45,46,48]. Here we used Evans seeds to provide a parallel determination of flux, and compared with the metabolomics and transcriptomics data.

**Figure 5:**
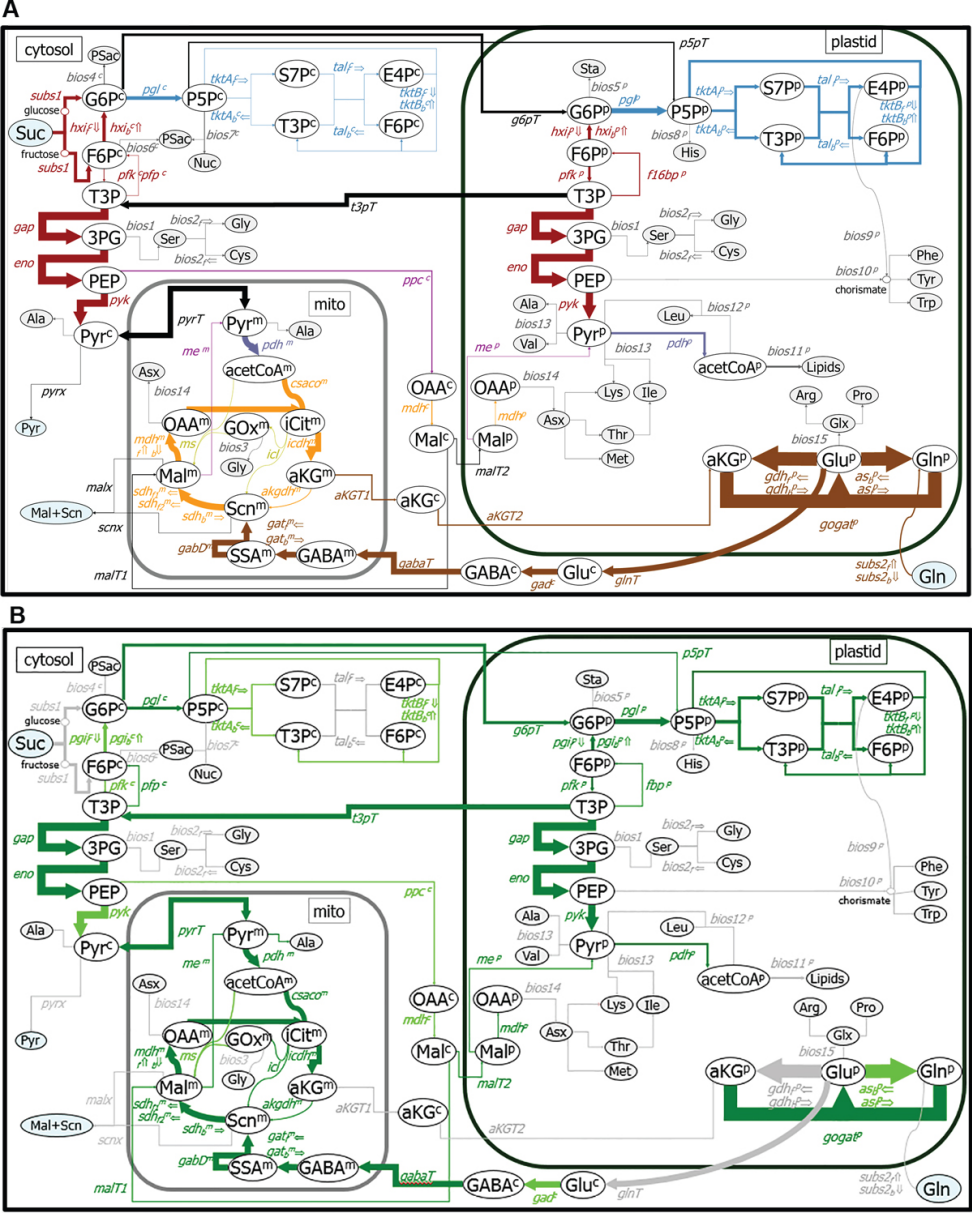
**Metabolic flux map of central carbon metabolism in cotyledons at early seed fill**. *Glycine max, var. Evans *plants were grown in a growth chamber. Seeds were harvested at 21 DAF and cultured in medium containing ^13^C-sucrose until 26 DAF. Widths of flux arrows are drawn in proportion to relative fluxes; arrow directions indicate net flux. Each flux is represented by the metabolic reaction, with a superscript indicating subcellular localization (if known): c, cytosol; p, plastid; and m, mitochondrion. Oval fill colors: intracellular metabolites (white); "sink" metabolites such as proteinogenic amino acids or polysaccharides (gray); metabolites absorbed from/discharged into the medium (blue). **A**. Metabolic flux map. Flux arrow and name colors: glycolysis and sucrose metabolism (dark red); pentose phosphate pathway (pale blue); TCA cycle (orange); pyruvate dehydrogenase (purple-gray); anaplerotic reactions (mauve); glyoxylate shunt (green yellow); glucose metabolism, GABA shunt and related transmembrane fluxes (brown); fluxes towards synthesis of biomass (gray); all other transmembrane fluxes (black). **B**. Integration of metabolic flux with transcriptomics data. Flux arrow and font colors: reactions that have transcript support for encoded proteins predicted to be in the same subcellular localization as the reaction (dark green); transcripts for encoded proteins of undetermined subcellular location (light green); no transcript identified from the soybean genome and/or and no transcript on the Affymetrix soybean genome chip, and/or multi-step reactions (grey).

The flux of metabolites was determined in isolated cotyledons in medium containing ^13^C [45,46] (Additional file 5: Tables S5a and S5b). The flux represents the pathway dynamics in the cotyledons at the quasi-steady-state developmental stage of early seed fill (21-26 DAF). The metabolic flux map representing central carbon metabolism is visualized in Figure 5A. The metabolic reaction network in early seed-fill soybean cotyledons consists of parallel pathways of glycolysis and oxidative pentose phosphate pathway (oxPPP) in the cytosol and the plastid. Flux through the γ-aminobutyric acid (GABA) shunt was quite high, as was the flux through glycolysis, the TCA cycle, and oxPPP. The high flux through the GABA shunt may reflect the need to shuttle glutamine and α-ketoglutarate between mitochondria (where the TCA cycle is located), and the plastids (the site of glutamine assimilation). Our analyses of the transcriptomics and metabolomics data identified pathways that were significantly changed during seed development (Additional file 3: Table S3) among those pathways identified as having a high flux. For example, in the transcriptomics data the TCA cycle and glycolysis were over-represented by Fisher's Exact test, with representative genes of these pathways decreasing during seed development (Clusters T5 and T6), and the MRPP test indicated these processes were significantly changed. Similarly, levels of metabolites in the TCA cycle and that of glutamate were altered. The flux through non-oxidative reactions of the oxPPP catalyzed by the transketolase (*tkt*) and transaldolase (*tal*) enzymes was much greater in the plastid as compared to the comparable cytosolic reactions. *F16BPase*, a key reaction of gluconeogenesis, was also higher in the plastid than the cytosol. The relative flux rate through a number of amino acid biosynthetic pathways (e.g., serine, glycine and cysteine) was comparatively low at this stage of seed development; this may indicate that these amino acids are formed predominantly via interconversion from other intermediates.

In most cases, the conclusions from the metabolic flux data were supported by the transcriptomics data (shown as green-color arrows in Figure 5B). For enzymes that are present in multiple subcellular compartments (e.g., cytosol, plastid, and mitochondrion), conclusions were supported by the transcriptomics data with the appropriate subcellular located isozyme-coding transcripts matching the flux analyses. The transcript levels of many genes of the GABA shunt, TCA cycle and glycolysis accumulated at far above median level; these transcript levels often reflect their metabolomic flux rate levels. For example, the flux through glutamate synthase (GOGAT) was about 21-fold greater than the flux through xylulose-5-P/P translocator (p5pT). Following the same trend, the level of the transcript encoding GOGAT was about 30-fold higher than the level of the transcript encoding p5pT.

### Integration of metabolomics and transcriptomics data in metabolic networking

**Figure 6:**
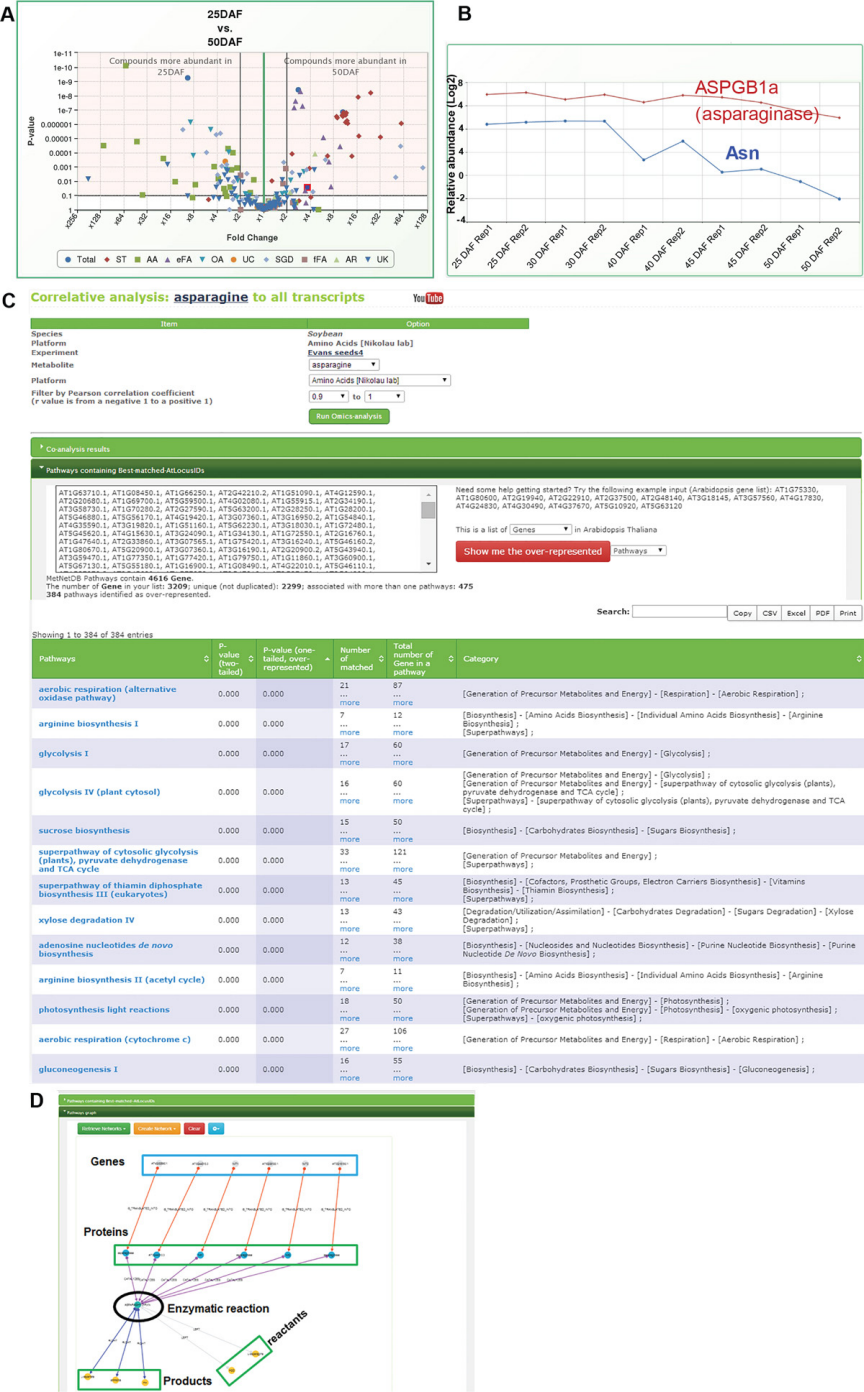
**Interactive analysis of metabolomics and transcriptomics data using PMR**. **A**. Volcano plot (p-value vs. fold change) comparison of metabolites in 25 DAF versus 50 DAF seeds in PMR http://metnetdb.org/PMR/. Clicking or mousing-over a metabolite brings up its annotation information. The data indicate that almost every amino acid is more abundant in 25 DAF seeds than 50 DAF seeds, while almost all sterols are more abundant in 50 DAF seeds than in 25 DAF seeds. **B**. Transcript Glyma05g07420.1 has a high correlation with asparagine accumulation during seed development (Pearson Correlation>0.86; p-value = 0.06). There are six genes in the soybean genome annotated as having sequence similarity to known asparaginase; of these, only Glyma05g07420.1 has an expression pattern similar to that of asparagine. These data indicate that Glyma05g07420.1 is a likely candidate for encoding the asparaginase activity associated with protein synthesis during seed fill. **C**. Other over-represented pathways in the set of genes that have a high correlation with asparagine accumulation. **D**. Asparaginase reaction in soybean visualized in PMR via Cytoscape [62].

We integrated these complex datasets to interpret metabolic changes associated with seed composition, highlighting as examples, the metabolism of asparagine, starch, and fatty acids. The amino acid asparagine is the major form of nitrogen imported from the vegetative soybean plant by the developing embryo and interconverted to other amino acids to support protein synthesis; it is therefore considered to be important to protein content in mature seeds [60]. We evaluated this aspect of metabolism using PMR. Volcano plots comparing the metabolomics of seeds at 25 and 50 DAF indicate that free amino acids are more abundant in 25 DAF seeds (also reported for several amino acids in Collakova *et al *[21]), while sterols are greatly increased in 50 DAF seeds (Figure 6A). As indicated by Figure 2C, Asn was the most abundant free amino acid in the developing Evans seeds but decreased quickly after 30 DAF. Co-analysis of the metabolomics and transcriptomic data indicated that Asn concentration varied during seed development and that this pattern strongly correlated with one of the six transcripts computationally predicted to encode an asparaginase-like gene (soybean locus ID: Glyma05g07420; Figure 6B). These data lead us to predict that the Glyma05g07420 gene encodes the particular asparaginase enzyme that is associated with amino acid interconversion during seed fill.

Further scrutiny of the over-represented pathways of the gene list identified by co-analysis of Asn to all transcripts (Pearson correlation 0.9-1.0; p-value < 0.05) (Figure 6C) revealed pathways involved in biosynthesis, respiration, regulatory and signaling pathways, and catabolism, particularly asparagine biosynthesis, were also over-represented. Figure 6D showed a pathway-graph for asparagine degradation from the over-represented pathways list in PMR. Co-analysis of Asn to all metabolites (Pearson correlation 0.88-1.0; p-value < 0.05) revealed amino acids, as well as lipid (such as trans-butenedioic Acid), and oligosaccharide etc.

**Figure 7:**
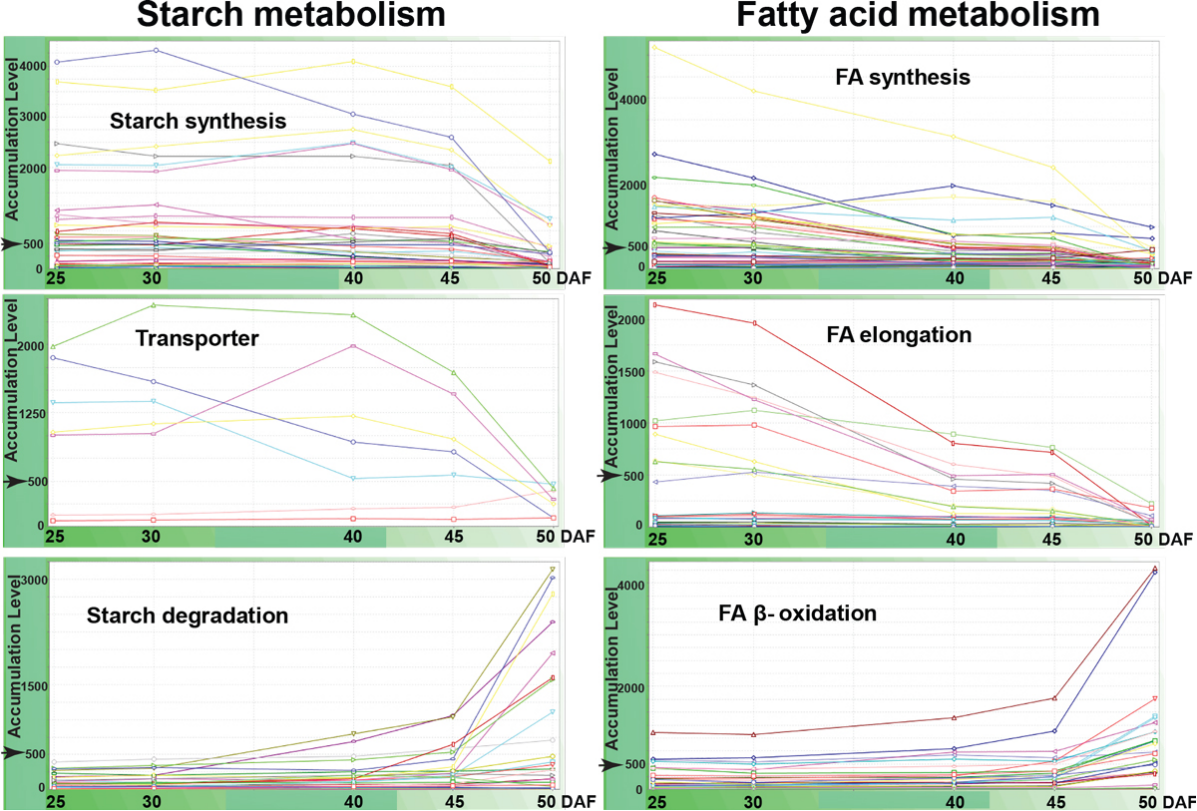
**Interactive bio-statistical integration of complex datasets reveal metabolic changes associated with seed composition**. Starch metabolism and fatty acid metabolism. The transcript levels for the genes in each pathway are visualized in MetaOmGraph http://www.metnetdb.org/MetNet_MetaOmGraph.htm. The data were normalized to a target signal intensity level of 500 for each chip.

Figure 7 illustrates a MetaOmGraph visualization http://www.metnetdb.org/MetNet_MetaOmGraph.htm of the levels of the transcripts known or postulated to be in starch and fatty acid metabolism during soybean seed development. Consistent with measured starch accumulation, which decreased rapidly after 45 DAF (Figure 1C and 1D), the levels of transcripts involved in starch synthesis and of those related transporters decreased after 45 DAF, while those involved in starch degradation had a rapid increase after 45 DAF. Expression of many postulated and known fatty acid metabolism genes was correlated with oil accumulation (Figure 1C and 1D). Oil increased steadily till 40 DAF, at which point oil content declined somewhat. A decrease in oil content at late seed maturation has also been reported previously in seeds of Arabidopsis and soybean [12,21]. The genes putatively involved in fatty acid synthesis and elongation decreased expression after 40 DAF, whereas genes involved in β-oxidation increased expression after 45 DAF. This may reflect, as has been shown for Arabidopsis [12], that during the later part of development, seeds prepare for the coming germination process when β-oxidation will be required to mobilize the stored oil reserves.

## Conclusions

The global profiling of genome expression during seed development provides the basis to formulate testable hypotheses regarding the function of genes and ultimately to identify the genetic regulatory networks that determine the structure and composition of the mature seed. Here, we detail the metabolomic and transcriptomic profiles of developing seeds of *Glycine max*, *var*. *Evans*, from early seed fill to maturation and desiccation. Furthermore, we illustrate the utility of **P**lant/Eukaryotic and Microbial **M**etabolomics Systems **R**esource (PMR). In part because it is an NoSQL environment, PMR provides rapid co-analysis of correlations with associated statistical determinations even for big data. PMR thus enables life scientists to explore metabolomics, transcriptomics datasets and to develop and extract biologically informed hypotheses from the data.

Soybean seed filling follows a developmental program in which protein and oil reserves increase, while starch levels decrease during seed development. Overall, the data substantiate the dynamic nature of seed development. During early seed fill, metabolic flux was greatest through the GABA shunt, glycolysis, the TCA cycle, and oxPPP. This is reflected in the transcriptomic and metabolomic data collected during seed development, with changes in metabolites and transcript levels being consistent with the changes in flux through these pathways. Interactive analysis of the transcriptomics data led to the identification of a transcription factor class, bHLH-Leu, that we postulate may participate in regulating the expression pattern of a co-expressed cluster of genes enriched in JA signaling and response motifs during soybean seed fill.

Our analyses indicate that during seed development modulations in end-products of metabolism are affected by a small proportion of the soybean genome, and that the majority of gene transcripts showed a relatively constant level of expression. In contrast, although the metabolite profiling is not as comprehensive as the transcriptomic profiling, the levels of almost every detectable metabolite are modulated during seed development. These metabolites are primarily the intermediates of the final storage reserves (i.e., intermediates of starch metabolism, and phospholipid and amino acid biosynthesis), but also includes metabolites that are unrelated to the seed reserves (e.g., sterol metabolism). These findings suggest that the metabolome is more sensitive to the developmental program than is the transcriptome. This is probably indicative of the fact that as genetic information is expressed through the processes of transcription and translation, coupled with the catalytic properties of the proteome, subtle changes at the transcriptome level are amplified at the level of the metabolome.

The combination of genetic resources, high-throughput experimental data and integrated bioinformatic analyses presented herein has enabled correlative-led depiction of complex programmed metabolic changes, which determine the composition of soybean seeds. The full transcriptomics and metabolomics data are freely available to researchers at PMR, http://www.metnetdb.org/pmr.

## Methods

### Plant growth and harvest

*Glycine max *variety *Evans *was grown in the field at the Bruner Research Farm near Ames, IA. Flowers at mid-plant nodes were tagged as they opened. Pods were harvested at 20, 25, 30, 35, 40, 45, and 50 DAF. Two replicate samples of three-seed pods were collected for each stage; 20 pods from five or six different plants were pooled for each replicate.

### Oil, protein, and starch determination

Oil was extracted from 100 mg freeze-dried tissue with 1 ml hexanes at 45°C for 60 min. The residue was pelleted by centrifugation at 5500 g for 10 min, and the extraction procedure was repeated four times. The pellet was retained for protein and starch analysis. The combined hexane-supernatants were pooled, dried at 60°C for 48 h. Soluble protein in the defatted pellet was precipitated 3 times in 1 ml of 240 g kg^-1 ^trichloroacetic acid and quantified by a Micro-Kjeldahl procedure [65]. Starch content was determined from 25 mg of the dried defatted pellet. Soluble sugars were removed by extracting the pellet with 4 treatments of 1 mL of 800 mL L^-1 ^ethanol heated at 60°C for 20 min, then centrifuged at 5500 g for 10 min. Starch in the final pellet was solubilized in water at 100°C and digested with amyloglucosidase according to Adams *et al *[66]. The liberated glucose was determined using a glucose oxidase technique [67] employing a microsample plate reader. Starch content was calculated as (mg glucose × 0.9).

### Plant material for transcriptomics and metabolomics

Pods were frozen immediately in *dry ice *and stored at -80°C until analysis. Frozen seeds were removed from the pods and selected visually for uniformity in size within each sample. The number of seeds extracted varied with seed development (7, 5, 4, 3, and 4 seeds were pooled at 25, 30, 40, 45, and 50 DAF, respectively).

### RNA and microarray analysis

RNA was extracted using the TRIzol RNA isolation protocol (Invitrogen Life Technologies, CA), and purified by Qiagen RNeasy Mini Kit (Qiagen, CA). Synthesis of labeled cRNAs, hybridization with Soybean Genome Arrays (Affymetrix, Santa Clara, CA), and scanning of the probe array were performed at the Iowa State University GeneChip Facility (Ames, IA). For data analysis, relative expression intensities were generated and normalized with Microarray Suite (MAS) 5.0 software (Affymetrix, CA) in the form of signal values. Global scaling (Affymetrix, CA) was used to normalize the data by adjusting the mean expression level of each chip to a value of 500.

The consensus sequences on the GeneChip® Soybean Genome Array downloaded from Affymetrix http://www.affymetrix.com/support were used to identify the Arabidopsis orthologs. The annotation is available in SoyBase http://soybase.org/AffyChip/AffychipAnnotation_Glyma1.1.txt?Submit=Submit.

### Soybean metabolomics

Four different analytical procedures were used to evaluate the relative abundance of metabolites in developing soybean seeds. Frozen seeds were initially pulverized into a powder while frozen in liquid N_2 _using a Freezer/Mill (SPEX SamplePrep, NJ). The powder was stored at -70°C until extraction.

Three of the four analytical procedures were targeted to specific class of metabolites (free amino acids, free fatty acids and phytosterols), the fourth was a non-targeted metabolite profiling procedure. A known weight of between 10 and 50 mg of pulverized soybean seeds were used in each analytical procedure. Details of each procedure are available at PMR http://www.metnetdb.org/pmr[37], as are the experimental data and metadata. We have previously used these methods to profile such metabolites in soybean [68], Arabidopsis [69-71], and *Hypericum spp *[72]. Combining the data from all these analytical platforms provided relative abundances of 338 analytes. Of these, 171 could be classified into seven chemically defined classes: free amino acids and amines (36 entities), aromatics (9 entities), esterified fatty acids (12 entities), free fatty acids (17 entities), organic acids (19 entities), sterols (30 entities), and sugars and derivatives (48 entities); an additional 167 analytes are chemically undefined (the eighth class of metabolites).

### Seed growth, harvest, and culture for metabolic flux analysis

The plants were gown, seeds were harvested, and cotyledons were cultured and analyzed as previously described [45]. Plants were grown in growth chambers under controlled conditions 27°C/20°C day/night, and 14-hour light. Pods were harvested 21 DAF. Four cotyledons (one from each seed) were isolated and cultured for six days. The medium was replaced after three days to ensure sucrose, the primary source of carbon to the cotyledons in planta, did not limit cotyledon growth *in vitro*. The flasks were shaken at 100 rpm, 26°C. Cotyledons were harvested after 6 days in culture for flux analyses. The absolute and relative metabolic fluxes were analyzed as described previously [45,46].

### Metabolic and transcriptomic data analysis

Metabolite accumulation levels were natural log transformed and median centered; similar data transformation was applied to Affymetrix signal values within each chip. These normalized log signals were analyzed separately for each metabolite/probe set using a linear model. Each linear model included fixed effects for replications and time points. As part of each linear model analysis, an overall F-test was conducted to scan for any change in mean accumulation/expression across the time points examined. The p-values from these F-tests were converted to q-values [59] to control FDR at specified levels.

### Cluster and over-representation analysis

K-medoids cluster analysis [73] was used to organize and visualize the accumulation patterns of the 273 metabolites that were above detection limits for at least one of the five time points during seed development. A similar analysis was also conducted for the 2,869 transcripts with q-values less than 0.01 in the overall F-test for change of expression over time. Euclidean distance between standardized accumulation/expression profiles was used to determine the dissimilarity between metabolites/genes when clustering. The number clusters were selected using the criterion described by Krzanowski and Lai [74], five for metabolites and eight for transcripts. The method of Nettleton *et al*. [61] and Fisher's exact test [56] were used to identify categories of genes/metabolites whose joint expression/accumulation distribution underwent changes during development. To determine over-representation of the transcripts and metabolites in the 351 pathways in MetNet and the transcripts in Gene Ontology categories, separate analyses for genes/metabolites were conducted for the pathways and for each of the three ontologies molecular function (1,053 categories), biological process (1,787 categories), and cellular component (381 categories).

### Systems bioinformatics analysis and data storage

MetNet systems biology tool suite http://www.metnetdb.org, http://metnetonline.org/index.php[49,50] for plant omics, and **P**lant/Eukaryotic and Microbial **M**etabolomics Systems **R**esource (PMR: http://www.metnetdb.org/pmr[37], were used for data deposition and statistical and interactive analyses of transcriptomics and metabolomics data. PMR currently contains multiple experiments from 26 species of plants and other organisms. Metabolomics or metabolomics/transcriptomics data can be submitted as private by any researcher for analysis; it is made public upon publication. PMR is platform-independent and species-independent. Because tens of thousands of data points are in each metabolomics/transcriptomics dataset, different researchers can explore diverse areas of metabolism and signal transduction within the same dataset. PMR stores metabolomics (and associated transcriptomics) data, evaluates data quality, compares metabolomes across biological samples, manages metadata, and co-analyzes metabolomics and transcriptomics data. Within PMR, metabolomics data can be compared across different biological samples by statistical analysis including t-test and principal components analysis. Computed values are stored graphically and presented using "volcano plots" [75]. PMR supports co-analysis of metabolomics and transcriptomics data from the same samples, allowing discovery of genes whose expression is associated with accumulation of specific metabolites; for co-analysis, correlation values is computed among metabolomics data, and between metabolomics and transcriptomics data.

TAIR 10 [76] identified 33,602 genes in *Arabidopsis thaliana*, with 41,671 gene models. The AraCyc-annotated database [77] consists of 521 pathways, with 3,325 enzymatic reactions, 8,846 individual proteins, 8,884 enzymes, and 2,614 metabolites. The original MetNetDB pathway database [49] was based on a traditional relational database management system (RDBMS). However, in order to traverse along a path in multiple pathways using a relational database, an RDBMS would suffer from the inefficiencies associated with multiple 'joining tables' which each may have >10,000 entities. To overcome this limitation of RDBMS, we employed the graph database, Neo4j [78,79], based on NoSQL to model the pathway database. Since the metabolic pathways and the chemical interactions can be represented into a directed graph, we established a graph-based MetNetDB, a web-based, computational frame-work that enables interactive visualization of metabolic and regulatory networks (Lee, Hur and Wurtele, unpublished). Networks can be retrieved as lists of such entities as genes, proteins, metabolites, and transcripts from the graph-based MetNetDB, and relationships among the entities can be represented as graphs. New features such as paths between two different entities (e.g., paths between a gene and a metabolite) can be obtained by applying graph theory. If a list of entities is too large to visualize, interpretable graph filters can be implemented to reduce the complexity and focus the list on the most likely pathways.

The *cis*-acting motifs in the gene promoters (1000 bp upstream of ATG) of the 2,869 transcripts in eight transcriptomics clusters, were evaluated using PLACE: A Database of Plant Cis-acting Regulatory DNA Elements http://www.dna.affrc.go.jp/PLACE/signalup.html.

### Availability of supporting data

The data sets supporting the results of this article are included within the article and its additional files. The metabolomics and transcriptomics dataset supporting the results of this article is available from PMR http://www.metnetdb.org/pmr; transcriptomics data are available online from the NCBI Gene Expression Omnibus http://www.ncbi.nlm.nih.gov/geo/ submission number GSE61350.

## List of abbreviations used

DAF, days after flowering; AA, amino acids and amines, AR, aromatics, eFA, esterified fatty acid, fFA, free fatty acid, OA, organic acid, SGD, sugars and derivatives (alcohols and acids), ST, sterols and tocopherols, UK, unclassified or unknown; Asn, asparagine; PMR, **P**lant/Eukaryotic and Microbial **M**etabolomics Systems **R**esource; MRPP, multiresponse permutation procedure; FDR, false discovery rate; RDBMS, relational database management system; GO, Gene Ontology; jasmonate, JA; GABA, γ-aminobutyric acid; oxPPP, oxidative pentose phosphate pathway.

## Competing interests

The authors declare that they have no competing interests.

## Authors' contributions

Conceived and designed the experiments: BJN, LL and ESW. Performed the experiments: LL, WZ, VI, MW, and CYD. Analyzed the data: ZS, DN, VI, JS, BJN, ESW and LL. Bioinformatics development: LL, ESW, MH, JL, ZA and NR. Contributed materials/analysis tools: MW. Wrote the paper: LL, BJN, and ESW. All authors read and approved the final manuscript.

## Supplementary Material

Additional file 1 **Table S1**. Metabolites, shown in Figure 2 and Table 1 grouped into five clusters based on co-accumulation patterns across the five time points of soybean seed fill. Click here for file

Additional file 2 **Table S2**. Transcripts, shown in Figure 3, which exhibited significant variation and were grouped into eight clusters based on co-accumulation patterns across the five time points of soybean seed development. Click here for file

Additional file 3 **Table S3**. Statistical analyses of the transcripts and metabolites in the co-expression clusters based on pathway membership and Gene Ontology terms. Click here for file

Additional file 4 **Table S4**. Statistical analyses of the transcripts in the eight co-expression clusters, based on promoter motifs. Click here for file

Additional file 5 **Table S5**. Metabolic flux analyses. Click here for file

## Declarations

This article has been published as part of *BMC Genomics *Volume 16 Supplement 3, 2015: Selected articles from the 10th International Symposium on Bioinformatics Research and Applications (ISBRA-14): Genomics. The full contents of the supplement are available online at http://www.biomedcentral.com/bmcgenomics/supplements/16/S3.
